# Long-term maintenance of *in vitro *cultured honeybee (*Apis mellifera*) embryonic cells

**DOI:** 10.1186/1471-213X-6-17

**Published:** 2006-03-17

**Authors:** Monica Bergem, Kari Norberg, Randi M Aamodt

**Affiliations:** 1 Department of Animal and Aquacultural Sciences, Norwegian University of Life Sciences, 1432 Aas, Norway

## Abstract

**Background:**

*In vitro *cultivation of cells allows novel investigation of *in vivo- *mechanisms and is a helpful tool in developmental biology, biochemistry and functional genomics. Numerous cell lines of insect species, e.g., silkworm and mosquito, have been reported. However, this is not the case for successful long-term cultivation of cells in honeybees.

**Results:**

Methods for cultivation of honeybee embryonic cells are discussed here. Pre-gastrula stage embryos were used to initiate cultures, and cells were reared on 96-wells microplates with Grace insect medium, supplemented with Fetal Bovine Serum. Cells proliferated in clusters, and maintained viable and mitotic for more than three months.

**Conclusion:**

We report here, for the first time, long-term cultivation of honeybee cells. Results represent a highly useful *in vitro*-system for studying a model organism of increasing importance in areas such as aging, sociality and neurobiology.

## Background

*In vitro *cultivation of cells of embryonic and somatic origin is a valuable technology for the exploration of *in vivo *mechanisms in organisms as diverse as plants and humans. Cells in culture have helped to attain a deeper understanding of functions and mechanisms conserved between organisms in evolution.

Cultivation of cells outside the organism enables study of developmental potential and differentiation [1,2], biochemical pathways [3] and genetic manipulation [4,5]. Cells can be maintained, some indefinitely, *in vitro *or be injected into living organisms for research or medical purposes. As the number of genomes sequenced has increased, the use of reverse genetics on cultured cells enables to address issues connected to genotypic-phenotypic relationships.

Primary cultures of embryonic cells have been used to establish cell lines in various insect species such as the fruitfly [6,7], fleshfly [8] and housefly [9]. As far as Hymenopteran species is concerned, though, there have been very few embryonic (as well as other) cell lines reported. Among the few, are cell lines from the pine sawfly *Neodiprion-Lecontei *[10] and the parasitoid wasps *Trichogramma pretiosum *[11] and *Mormoniella vitripennis *[12]. Embryonic cell lines, such as the Drosophila S2 and Kc, are widespread and currently used extensively within a number of research areas [3].

The honeybee (*Apis mellifera*) is an increasingly important model organism for research on aging [13], social behaviour [14] and neurobiology [15]. It's genome has recently been sequenced and will provide new insight into the genetics of honeybees, as well as comparisons with other species. Establishment of long-term cell cultures will enable to study several of these subjects in addition to e.g. RNA interference, at the cellular level. However, despite a pressing need, there are currently no protocols for long-term maintenance of honeybee cells available. There are, however, reports on short-term cultures [16-18] but, to our knowledge, there are no publications on how to maintain honeybee cells in culture for more than a month.

The honeybee embryo is about 1.6–1.8 mm long, with a maximum diameter of 0.35 mm [19]. In the course of 72 hours from oviposition to hatching, embryonic development occurs through 10 developmental stages [20-22]. The cellular blastoderm is formed at stage 3, about 9 hours after oviposition (h.a.o.), but until stage 6, approximately 33 h.a.o., the blastoderm cells are not completely separated. This stage is followed by gastrulation with migration and subsequent differentiation of cells.

Long-term cell cultures would also be especially valuable in studies of honeybee pathology. The honeybee colony is under considerable pathogenic pressure, and an improved understanding of the interaction between honeybee cells and viruses such as Deformed Wing virus (DWV) and Sacbrood virus (SBV), as well as other intracellular pathogens, would be highly valuable and commercially important.

Another application of cells in culture is as donors in cell transplantations for cell-mediated gene transfer and production of chimeras. Our group has recently successfully produced chimeras by transplantation of cells between embryos [23]. Access to long-term cultures of highly potential embryonic cells will give new opportunities for studies and transplantation of labelled and modified cells.

Here, we report methods for establishment of long-term cultures of honeybee embryonic cells. We have initiated cell cultures from honeybee embryos at various developmental stages, cultivated under various growth conditions. This is the first time honeybee cells have been reported to survive in culture for more than one month. Undifferentiated cells from pre-gastrula stage embryos were successfully cultured for more than three months.

## Results

More than 150 cultures were initiated and various procedures and conditions were explored during the course of the work. The protocols outlined below were found to support cells to remain mitotically active, morphologically undifferentiated and viable for more than three months.

### Media and growth conditions

Grace insect media (supplemented with L-amino acids), supplemented with 15 % FBS and 1.5 % Gentamycin, final pH 6.3, was found to support long-term growth of honeybee embryonic cells most successfully. We could not see a difference in growth between cells cultivated in Leibovitz's L15 or in the medium developed by Kreissl and Bicker [17], but both provided poorer growth conditions than Grace.

It was advantageous to cultivate cells in 200 μl medium, on 96-well, uncoated micro plates. Cells incubated at 30°C had successful growth. Evaporation of media was best avoided by wrapping plates in two layers of plastic sheets.

**Figure 1:**
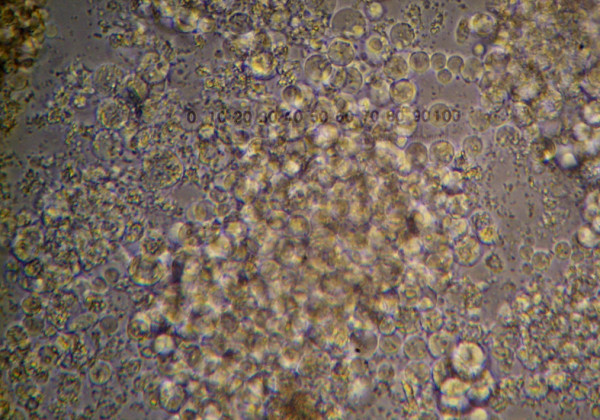
**Honeybee embryonic cells**. Embryonic cells (36–40 h.a.o.) grew as single cells or in large clusters. Image captured 2 weeks after initiation. 400 × magnification.

Medium replacements were every fourth day, by replacing 3/4 of the spent medium with an equal volume of fresh, prewarmed medium. Cells grown under the conditions suggested, formed cell aggregates of various sizes (Fig. 1) present throughout the culture, in agreement with reports on embryonic cell cultures of other species [9,24,25].

Makisterone, an insect growth-promoting hormone [26,27], had no effect on cell proliferation, but no deleterious effects were registered either. The same was true for various dosages of supplemented hemolymph, which is often recommended as supplement in culture media in order to reproduce as closely as possible the norms of the natural physiological environment of the cells [6].

### Viability

**Figure 2:**
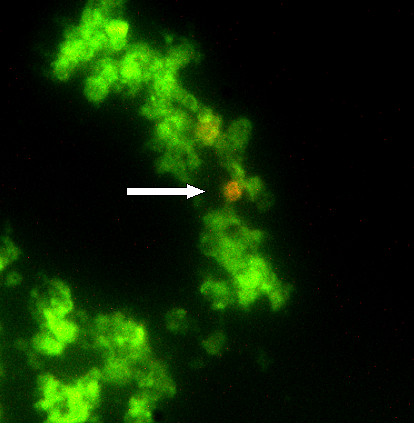
**Distribution of viable and dead cells**. Viable cells appeared fluorescently green under  UV-light, whereas dead cells were red (white arrow). Image captured from a 4-week old culture, initiated from embryos 36–40 h.a.o.. 400 × magnification.

The Live/Dead Viability kit (Calcein-EthD counter stain) and the Vybrant™ CFDA SE Cell Tracer Kit both provided information on viability of the cultures cells. A working solution of 4 μM calcein AM and 2 μM EthD-1, was found to be optimal for the staining procedure. Exact ratios between living and dead cells were not assessed, but only a small fraction of the adherent cells in the culture were dyed red (dead), as determined by repeated visual observations (Fig. 2). The microscopic studies had to be performed quickly (within 1 hour), as an increasing amount of cells were dyed red by ethidium, despite having already obtained green appearance. This was probably due to the strain the procedure conferred to the cells, causing them to die and then dye.

**Figure 3:**
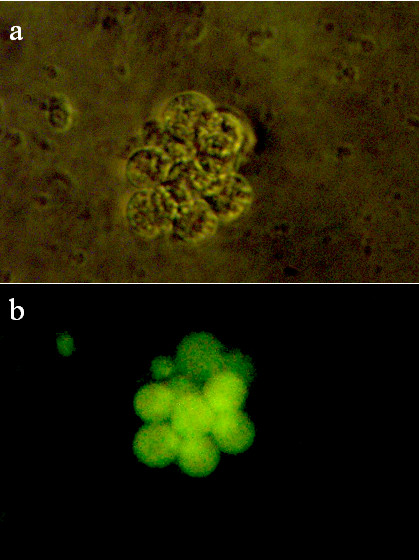
**Viability of cells studied by use of cell tracer**. Viable cells (a) dyed by CFDA SE cell tracer, obtained a green fluorescent appearance (b), which were maintained for more than one month. 400 × magnification.

For CFDA SE, we found that adding 100 μl of 10 μM CFDA SE provided optimal staining. This dye was non-toxic and maintained fluorescence within cells for over 30 days (Fig. 3).

### Cell proliferation

**Figure 4:**
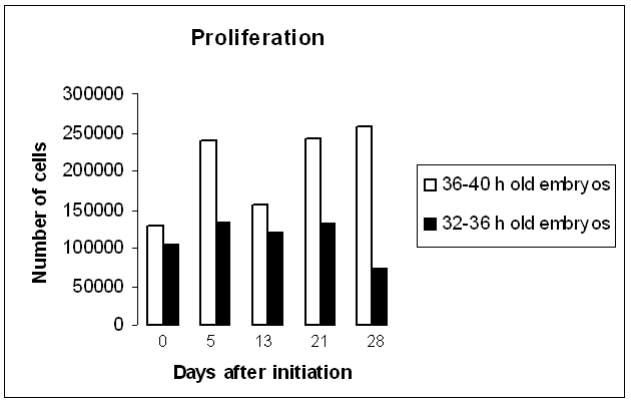
**Estimation of proliferation**. Proliferation was estimated, based on cell counts over 28 days, in cell cultures initiated from 15 embryos, 32–36 h.a.o. and 36–40 h.a.o.. Cell cultures initiated from embryos 36–40 h.a.o. showed higher proliferation than the cultures initiated from embryos 32–36 h.a.o.

**Figure 5:**
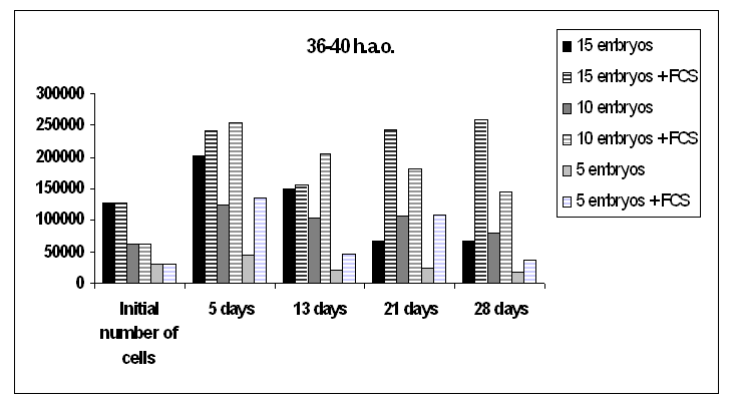
**The effect of variables on proliferation**. Cell counts from 36–40 h.a.o. cell cultures, with varying number of initial eggs and with/without FBS. X-axis displays number of days after initiation of cultures, y-axis displays number of cells. Two cultures were initiated on the same number of embryos, whereupon one was added FBS (5, 10 or 15 %) whereas the other were not. Cultures initiated from 15 embryos, supplemented with FBS (15 %) were most successfully maintained. Generally, cultures which were reared in medium with FBS achieved higher proliferation rates.

To determine cell proliferation rates under various conditions, selected cell cultures were counted at the day of initiation and followed up for several weeks. The following factors were varied: 1) age of initial embryos (32–36 h.a.o, 36–40 h.a.o.), 2) number of embryos used to produce the cultures and 3) medium with or without FBS. The highest proliferation rates were obtained for cultures initiated from embryos 36–40 h.a.o. (Fig. 4). The most successfully maintained and mitotically active cultures were those initiated from 15 embryos and clearly, the supplementation of FBS to cultures generally produced better proliferation rates in all cultures studied (Fig. 5)

**Figure 6:**
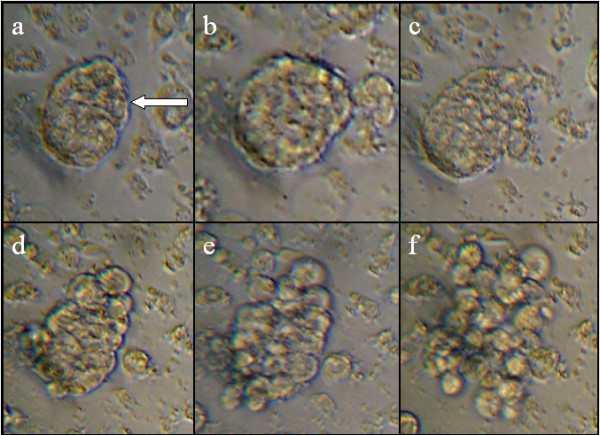
**Proliferation in cell clusters**. Proliferation occurred in cell clusters (a) arrow) as shown by time laps pictures, a) initial time, b) after 7 hours, c) after 20 hours, d) after 28 hours, e) after 32 hours and f) after 44 hours. 400 × magnification.

Proliferation occurred within cell aggregates as seen in Fig. 6, or by separate cells in areas with high cell density, possibly suggesting that mitagenic factors are secreted by the cells themselves. Although mitotic frequencies were low, cells were highly viable and we observed a dynamic development in varying properties related to adherence and cell number. Cultures that reached confluence were passaged. In most cultures, cells were still proliferating after three months although at varying rates.

### Cell characterisation

Immediately after initial cultivation, cells from embryos of all ages except for embryos, 16–18 h.a.o., were large (20–25 μm) and round. Cells from 16–18 h old embryos were pear shaped with a seemingly ruptured membrane at the narrow end. Most cells became somehow adherent during the first 24 hours, but were easily loosened from the base of the well by use of a pipette tip. We believe most cells were semi-adherent, as we could repeatedly observe floating cells and aggregates near the well bottom and the loose connection of stationary cells to the base. Dead cells were likely to loose adherence and be pipetted off during replacement of medium.

In cultures grown with FBS, cells often clustered together in small colonies, in which cells underwent mitosis. In such environment, cells remained large and round and actively proliferating for more than three months. In cultures without FBS, cells underwent a change in morphology after the first week, capturing a wrinkled shape and smaller size (5–15 μm). These cells were nevertheless also viable and mitotic for more than three months.

After 10 weeks, the number of cells eventually declined in most cultures, independently of the various factors.

*16–18 h.a.o.*: Cells were pear-shaped at initiation, changing later to a rounder and smaller shape (7–15 μm) with rough cell membrane. Some cultures were, for reasons unknown, more mitotically active than others and were passaged up to five times during a 3-months cultivation. With these exceptions, proliferation was generally slow. Addition of FBS (5–15 %) or various amounts of hemolymph had no effect on proliferation. Cells showed no sign of differentiation.

*30–32 h.a.o.*: Cells were round and large at initiation, but changed in morphology after one week and became smaller, with wrinkled cell membranes. When FBS was added, the cells remained round for a longer time, before eventually reaching a similar morphology as cells reared without FBS. They were nevertheless mitotic and viable, mainly occupying outer areas of the wells. Mitosis occurred in areas with high cell density.

*32–36 h.a.o.*: Cells remained round and large, in FBS-media. We did not observe changes in morphology. Proliferation occurred in clusters and in areas of high cell density.

*36–40 h.a.o.*: Cells remained round and large when grown in FBS media (Fig. 6). There was a constant mitotic activity inside and outside cell clusters. Cells did not show sign of differentiation.

**Figure 7:**
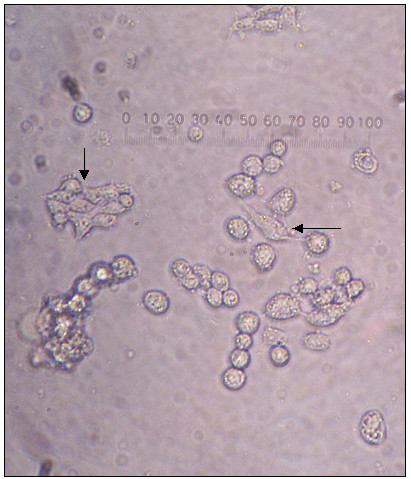
**Differentiation of cells initiated from embryos 36–44 h.a.o..**. Cultivated cells from embryos 36–44 h.a.o.. Some cells obtained a differentiated, elongated morphology, unlike cells in cultures from younger embryos. 400 × magnification.

*36–44 h.a.o.*: Initially, most cells were round and large; however, after only a few days, small groups of cells captured a more spindle shaped morphology and were seemingly differentiating (Fig. 7). We stained these cells with cell tracer, which proved the cells were viable (not shown).

### Microscopic studies of pre-gastrula stage embryos

**Figure 8:**
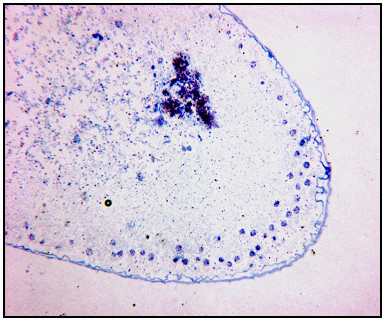
**Longitudinal section of embryo at 32–34 h.a.o..**. A fixed and sliced embryo (32–34 h.a.o.) demonstrate the location of cells at the time of cultivation; near the embryo periphery.

Longitudinal sections were prepared of fixed and sliced eggs at age 32–34 hours, to determine the cellular composition of the egg interior. As shown in Fig. 8, at this stage cells were mainly located at the periphery of the embryo, as described by DuPraw [19].

## Discussion

Although there are several reports on short-term cultivation of honeybee cells, long- term maintenance seems to remains an unsolved problem [28]. Reports on cultivated honeybee cells include hemocytes [16], neurons [17,29], antennal cells [18] and embryonic cells [30], but not pertained to long-term. Our results demonstrate that, under the appropriate circumstances, embryonic cells can be cultivated for more than three months, and may constitute an *in vitro *system for studies and manipulations of the honeybee.

Long-term cell cultures will certainly increase the value of the honeybee as a model organism; enabling new opportunities for studies of various research subjects, including host-parasite relationships, use of cells as donors in cell transplantations for cell-mediated gene transfer, and production of honeybee chimeras.

Our studies during the last three years, provides new knowledge about how to initiate and maintain honeybee embryonic cells in culture. We found that the medium able to support long-term cultivation was less complex than first expected. The most successful growth and maintenance was achieved in the commercial medium Grace insect medium, supplemented with 15 % FBS and antibiotics. Grace contains insect-supporting compounds, including yeastolate and lactalbumine hydrolysate, both additives known to be growth-promoting substances in insect cell cultures [31]; the medium has been reported to successfully support other insect cells *in vitro *[32,33].

So far, we have tested only a fraction of the numerous commercial insect media available, and further exploration of various media and supplements may contribute to develop even more favourable conditions to the cells, as utterly increased proliferation rates would be beneficial in many cases.

In addition to studying the supplements FBS and hemolymph, we tested makisterone as growth promoting additive, but we found no positive influence on proliferation of embryonic cells of our species. The same applied to addition of hemolymph, whereas FBS, in contrast, had a measurably positive influence on proliferation and on maintenance of initial morphology. There are numerous reports on factors with stimulating effects on proliferation of insect embryonic cells, such as chicken egg ultra filtrate [34] and sapecin [35]. Additionally, media, originally designed for culture of honeybee organs [36,37], may also provide appropriate growth conditions for cells. However, we did not examine these in our study.

Our results suggest that initiation of honeybee embryonic cell cultures should be performed at pre-gastrula stage. At late stage five, early stage six, the final separation of the blastoderm cells is completed [19,22] and the cells are provided with distinct membranes, enabling their maintenance as units in an *in vitro *environment. Cells of younger embryos were more likely to disrupt during the dissociation procedure, in agreement with observations reported by Cross and Sang [38], in their work with embryonic cells of *Drosophila*.

We found that cells from eggs 36–40 h.a.o. produced the most successful cultures. Cell from eggs at this stage remained mitotic, viable and seemingly undifferentiated, with an unaltered morphology until experiments were terminated. It is likely that cells from this stage can be used in future developmental studies, for example, of differentiation. However, such studies requires tissue-specific differentiation factors, which, as far as we know, there is currently no access to.

Cells from eggs at earlier stages either lacked complete cell membranes or were highly sensitive to the *in vitro *environment; they had a shrunken morphology and low proliferation rates. This was particularly true for the 16–18- and 30–32-groups, whereas in the 32–36-group the cells maintained a round shaped morphology, but proliferation was lower than for the 36–40-group. Cell cultures from the oldest eggs (36–44 h.a.o.) diverged from the rest of the cultures by having cells that appeared differentiated. These cultures were mixtures of round and spindle shaped cells, not found in cultures of younger eggs. These observations were not surprisingly though, considering the developmental stage of these eggs (early gastrulation). Interestingly, and in contrast to cultures from older embryos, FBS had no effect on cultures initiated from early embryos (16–18 h.a.o.), which may suggest that cells at this early stages cannot utilize FBS, either in matter of lacking receptors or even metabolic pathways.

Longitudinal observations of cultivated cells showed that proliferation occurred mainly in cell clusters. A 44-hour microscopic study of such clusters indicated that the cells divided inside the aggregate, followed by a "release" of daughter cells. The same was observed during studies of housefly embryonic cell cultures [9]. We found that, the pattern observed was seen mainly in cells initiated from eggs older than 32 h.a.o., whereas, in the younger cultures, mitosis occurred outside clusters, although, primarily in areas of high cell density. These observations suggest that the cells produce and secrete mitagenic substances that trigger proliferation in neighbouring cells.

Our observations of cell cultures stained with calcein and ethidium showed that the large majority of cells were viable, suggesting that dead cells loose attachment and floats into the medium. These cells would then be removed during the change of medium. This suggestion is also supported by our observations over time, where mainly mitotic cells were seen, whereas areas of older and dead cells disappeared.

It is likely, however, that both viable and dead cells were pipetted off during media replacements and, certainly, these cells could have been collected by centrifugation. However, there was no way of distinguishing viable cells from dead at this point, thus, we chose to discharge these withdrawals.

Our initial motivation for studying embryonic cells and for developing protocols for their culture was mainly to create a system allowing maintenance and manipulation of cells for subsequent injections into honeybee embryos. We have recently produced chimeric honeybees by cell transplantation between embryos [23]. Establishment of protocols for long-term cultivation of embryonic cells proves new opportunities for study of chimerism in bees.

The protocols discussed can be used in various types of studies. Primary cultures, as reported here, can be used to create continuous cell lines, as for the fruitfly (*Drosophila melanogaster*) [7], fleshfly (*Sarcophaga peregina*) [8] and housefly (*Musca domestica*) [9].

Cell lines from various tissues in numerous insect species have been utilized in many areas of biological research, including physiology, toxicology and pathology [39]. Cultivation of embryonic cells provided useful insights into early mechanisms of embryonic development, differentiation and cell fate and, under appropriate conditions, larval and adult cells of mesodermal, ectodermal and endodermal origin can develop *in vitro *from such cell lines [40].

Cell cultures can also be used in studies of pathogen-host relationship [41,42], e.g., involving viruses and intracellular parasites. There are 18 reported viruses associated with the honeybee [43], of which at least 6 have been sequenced [[44-47], GenBank Accession no. NC-004830 and NC-004807]. The most relevant ones are viruses DWV, acute bee paralysis (ABPV), slow paralysis virus (SPV), Kashmir bee virus (KBV) and cloudy wing virus (CWV), which are associated with the varroa mite (*Varroa destuctor*), known to cause depletion of honeybee colonies. Also, an *in vitro *system would be highly valuable for studies of parasites such as Nosema (*Nosema apis*), which reproduce in epithelial cells in the honeybee midgut. A report on infection of DWV on cultivated embryonic cells is forthcoming (Bergem and Forsgren, manuscript in prep.).

For the cell cultures reported here, we see also interesting potential in reverse genetics, as the honeybee genome has been sequenced, and RNA interference has already proved to be a successful tool for *in vivo *studies of gene function and of phenotypes [48,49].

## Conclusion

The work provided here demonstrates the first reported long-term *in vitro *cultivation of honeybee embryonic cells. We have developed methods to initiate cell cultures from pre-gastrula stage honeybee embryos and were able to successfully cultivate viable and mitotically active cells for more than three months. As the honeybee is becoming an increasingly important model organism, long-term cell cultures introduce a useful system to study new and interesting subjects within research areas, such as developmental biology, pathology and reverse genetics.

## Methods

Honeybee cells were cultivated from embryos at five different stages; 16–18, 30–32, 32–36, 36–40, 36–44 h.a.o. Investigations were carried out to find appropriate growth requirements, including growth media and supplements, basal coating and number of embryos to initiate cultures.

### Initiation of cell cultures

Eggs were collected from egg collection hives [50], incubated at 35°C until appropriate age, and subsequently removed from cell cups into eppendorf tubes. 1.2 ml of 2.5 % potassium hypoclorite was added for 10 min, to sterilize the egg surface and to weaken the chorionic membrane [19]. Following 3 continuous washes with dH_2_O, 200 μl of freshly prepared medium was added and cells were released into the medium by crushing eggs with a 200 μl pipette tip. After 5 min centrifugation at 2000 rpm, supernatant was removed, cells were resuspended in 200 μl of medium and each cell suspension was transferred into separate microplate wells. Cells were incubated at 30°C [51,33].

### Media and growth conditions

Grace Insect Medium Supplemented (with L-amino acids, Gibco, Invitrogen, UK) is a serum-free medium, adapted to the requirements of insect cells, containing growth promoting Lactalbumin Hydrolysate and Yeastolate. To determine the optimal media composition, were added, in various combinations and dosages, the supplements FBS (Gibco, Invitrogen, UK) in concentrations of 5–15 %, the honeybee 20-Hydroxyecdysone-equivalent, Makisterone A (A.G. Scientific, Inc.) to 1–5 ng/μl of final concentrations, and hemolymph to 2–4 % of final concentration. 0.5 % of Gentamycin was added all media compositions.

Cells from all "age groups" were also cultures in Leibovitz's L-15 (Gibco, Invitrogen, UK) as well as a medium reported by Kreissl and Bicker [17].

Supplementary hemolymph was collected from nurse bees and added directly to Grace medium on ice. Hemocytes were spinned down at 5000 rpm 5 min before removal of haemolymph-containing supernatant. Supernatant was filtered by use of 0.22 μm Millex-GV filter units (Millipore), before added.

Cells were objected to growth in 6, 24 and 96 wells plates (Falcon). To investigate the effect of coating of the culture dishes, we examined BD Cell-TAK™ (BD Biosciences). 96-well plates were coated and used to culture cells as described above.

### Viability

Viability of cells was assayed by calcein in combination with ethidium, counterstaining (Live/Dead Viability kit, Molecular Probes). Dye was added, either directly to colonies in wells, or to extracted cell samples. For direct staining in wells, we grew cells on chamber slides (Lab-Tek), to facilitate subsequent microscopic studies. Viewed by fluorescence microscopy, nuclei of dead cells were stained by ethidium (red), whereas cytoplasma of viable cells were stained by calcein (green). Dosages were applied as recommended by manufacturer.

A second staining kit, the Vybrant™ CFDA SE Cell Tracer Kit (Molecular Probes) was also used to confirm viability. The following dosages of dye was added to cell samples; 0.5, 5.0, 7.5, 10.0, 12.5, 15.0, 25.0 μM, to find the optimal working solution.

### Proliferation

To evaluate growth pattern and proliferation, cells cultured under various conditions were collected and counted every 7^th ^day. Cells were stained by tryphan blue (0.02 %) and counted by use of haemocytometer.

### Microscopic studies of pre-gastrula stage embryos

To study the distribution of cells within embryos, 32–34 hours old eggs were fixed in 4 % formaldehyde solution (methanol-free), supplemented with 0.2 % Triton X-100 (Sigma). Before embedding, eggs were washed thoroughly in 0.05 M PIPES buffer (Sigma) (3 × 15 min). Eggs were dehydrated in a graded series of ethanol (70, 90, 96, 100 %) and infiltrated in graded series of LR White (Chemie-Teknik AS) (LR White: EtOH, 1:3, 1:1, 3:1,1:0). Thereafter, they were polymerised in 60°C over night in embedding moulds. One-micron slices were cut on Reichert-Jung ultramicrotome, contrasted with Stevenell's solution and mounted in DePex (Chemie-Teknik AS), before being examined with Leica DMLB microscope. Longitudinal sections were studied, to investigate the distribution of cells within pre-gastrula stage embryos.

## Authors' contributions

MB was responsible for the experimental design, performance of experiments, analysis and interpretation of data and writing of manuscript. KN made considerable contributions to the development of protocols. RA critically read manuscript drafts. All authors approved of final manuscript.
